# Prevalence and patterns of rifampicin and isoniazid resistance conferring mutations in *Mycobacterium tuberculosis* isolates from Uganda

**DOI:** 10.1371/journal.pone.0198091

**Published:** 2018-05-30

**Authors:** Edgar Kigozi, George W. Kasule, Kenneth Musisi, Deus Lukoye, Samuel Kyobe, Fred Ashaba Katabazi, Eddie M. Wampande, Moses L. Joloba, David Patrick Kateete

**Affiliations:** 1 Department of Immunology and Molecular Biology, School of Biomedical Sciences, Makerere University College of Health Sciences, Kampala, Uganda; 2 Department of Medical Microbiology, School of Biomedical Sciences, Makerere University College of Health Sciences, Kampala, Uganda; 3 National Tuberculosis Reference Laboratory, Kampala, Uganda; 4 National Tuberculosis/Leprosy Program Ministry of Health, Kampala, Uganda; 5 College of Veterinary Medicine, Animal Resources and Biosecurity, Makerere University, Kampala, Uganda; Indian Institute of Technology Delhi, INDIA

## Abstract

**Background:**

Accurate diagnosis of tuberculosis, especially by using rapid molecular assays, can reduce transmission of drug resistant tuberculosis in communities. However, the frequency of resistance conferring mutations varies with geographic location of *Mycobacterium tuberculosis*, and this affects the efficiency of rapid molecular assays in detecting resistance. This has created need for characterizing drug resistant isolates from different settings to investigate frequencies of resistance conferring mutations. Here, we describe the prevalence and patterns of rifampicin- and isoniazid- resistance conferring mutations in isolates from Uganda, which could be useful in the management of MDR-TB patients in Uganda and other countries in sub-Saharan Africa.

**Results:**

Ninety seven *M*. *tuberculosis* isolates were characterized, of which 38 were MDR, seven rifampicin-resistant, 12 isoniazid-mono-resistant, and 40 susceptible to rifampicin and isoniazid. Sequence analysis of the *rpoB* rifampicin-resistance determining region (*rpoB*/RRDR) revealed mutations in six codons: 588, 531, 526, 516, 513, and 511, of which Ser531Leu was the most frequent (40%, 18/45). Overall, the three mutations (Ser531Leu, His526Tyr, Asp516Tyr) frequently associated with rifampicin-resistance occurred in 76% of the rifampicin resistant isolates while 18% (8/45) of the rifampicin-resistant isolates lacked mutations in *rpoB*/RRDR. Furthermore, sequence analysis of *katG* and *inhA* gene promoter revealed mainly the Ser315Thr (76%, 38/50) and C(-15)T (8%, 4/50) mutations, respectively. These two mutations combined, which are frequently associated with isoniazid-resistance, occurred in 88% of the isoniazid resistant isolates. However, 20% (10/50) of the isoniazid-resistant isolates lacked mutations both in *katG* and *inhA* gene promoter. The sensitivity of sequence analysis of *rpoB*/RRDR for rifampicin-resistance via detection of high confidence mutations (Ser531Leu, His526Tyr, Asp516Tyr) was 81%, while it was 77% for analysis of *katG* and *inhA* gene promoter to detect isoniazid-resistance via detection of high confidence mutations (Ser315Thr, C(-15)T, T(-8)C). Furthermore, considering the circulating TB genotypes in Uganda, the isoniazid-resistance conferring mutations were more frequent in *M*. *tuberculosis* lineage 4/sub-lineage Uganda, perhaps explaining why this genotype is weakly associated with MDR-TB.

**Conclusion:**

Sequence analysis of *rpoB*/RRDR, *katG* and *inhA* gene promoter is useful in detecting rifampicin/isoniazid resistant *M*. *tuberculosis* isolates in Uganda however, about ≤20% of the resistant isolates lack known resistance-conferring mutations hence rapid molecular assays may not detect them as resistant.

## Introduction

Tuberculosis (TB) is a disease of global public health importance declared the deadliest infectious disease alongside HIV/AIDS [1–3]. Although new cases have been reducing every year [3, 4], TB has persisted in human populations and it remains among the top 10 causes of death worldwide [3, 4]. In 2014 there were 9.6 million TB cases reported and 1.5 million deaths; more than 95% of the deaths occurred in developing countries [3, 5].

TB control programs in many countries rely on rapid identification of cases for effective treatment of the disease. Importantly, the drug-resistant forms of TB require accurate diagnosis of the type of resistance to guide therapy and interrupt transmission of resistant organisms in communities [6]. However, the conventional culture-based diagnostics for TB are slow, labor intensive and expensive, and they are often not available in the developing countries. The demand for rapid TB diagnostics led to the development and introduction of commercial molecular tests into practice. Two commonly used tests in Uganda are the “Xpert MTB/RIF” (GeneXpert, Cepheid Inc.), which diagnoses TB and detects rifampicin resistance [7–11], and the GenoType MTBDRplus (line probe assay [LPA] from Hain Life Sciences Nehren, Germany), which detects *Mycobacterium tuberculosis* and its resistance to both rifampicin and isoniazid [12–14]. Rifampicin and isoniazid are the most important anti-tuberculosis drugs, and resistance to both drugs is commonly referred to as “multidrug resistance” (MDR-TB) [1]. Importantly, although the commercial molecular tests rapidly and accurately diagnose TB [15, 16], they may not detect all the resistance especially in strains with novel or unknown resistance mechanisms. The molecular tests are also ineffective at detecting heteroresistance (“the occurrence of populations of both drug-susceptible and drug-resistant isolates within the same clinical sample” [17]) due to mixed-strain TB infections. This has led to reports of varying efficiencies in the performance of rapid molecular tests in detecting drug resistance, depending on the drug in question [6, 18], or location of the strains under study. Without doubt, differences in geographical distribution of TB strains (i.e. *M*. *tuberculosis* lineages and/or sub-lineages/genotypes) across continents [6, 18–21] significantly affect the performance of rapid molecular tests [22–24].

Molecular tests for TB target spontaneous point mutations in specific genes and/or loci in the *M*. *tuberculosis* chromosome that are associated with drug resistance [25], and only a limited number of mutations accounts for majority of the resistance to anti-TB drugs [6, 18]. Rifampicin-resistance, which is the best understood of all anti-TB drugs, is associated with mutations in an 81 base pair region of the *rpoB* gene commonly referred to as the rifampicin-resistance determining region (*rpoB*/RRDR). Mutations within the *rpoB*/RRDR confer resistance to rifampicin in majority of the rifampicin-resistant isolates [6], and three key mutations i.e. Ser531Leu, His526Tyr, and Asp516Val are reported to be predominant worldwide [6, 26, 27]. For isoniazid, mutations mainly in *katG* and *inhA* gene promoter, and infrequently *ahpC*, *oxyR*, *kasA*, *furA* and *ndh* genes, confer resistance to the drug [6, 18]. Approx. 64% of the phenotypic resistance to isoniazid globally is attributed to the *katG*/Ser315Thr mutation [18].

Regional variation in the frequencies of rifampicin and isoniazid resistance-conferring mutations has been reported and this could limit the sensitivity of molecular tests in detecting resistance [6, 18]. As such, it is becoming increasingly clear that the performance of rapid molecular tests in TB cannot be extrapolated from one setting to another, but needs to be validated in each geographic setting [23, 24], through systematic surveys to ascertain the mutation profiles and frequencies in geographic regions where the tests are being deployed [6, 18, 20]. It is through such investigations that the performance of new genotypic tests for TB in particular settings can be assessed. DNA sequencing is presumed to be the most appropriate approach for elucidating the patterns and frequencies of resistance conferring mutations in *M*. *tuberculosis*, and hence, evaluating the performance of new genotypic tests for TB [6].

Here, we describe the frequencies, patterns and distribution of rifampicin/isoniazid-resistance conferring mutations in MDR-TB isolates from Uganda based on sequence analysis of the *rpoB*/RRDR, *katG* and *inhA* gene promoter. We demonstrate that sequence analysis of those fragments is useful in detecting rifampicin- and isoniazid-resistance in Uganda however, a significant number of MDR-TB isolates lacks known resistance-conferring mutations implying that rapid molecular assays will miss detecting them as resistant to the drug in question. Also, we describe key associations between the circulating *M*. *tuberculosis* genotypes and occurrence of rifampicin/isoniazid resistance conferring mutations, providing new insight as to why the predominant TB strain in Uganda is weakly associated with MDR-TB.

## Results

### Frequency of *M*. *tuberculosis* genotypes

We investigated 38 MDR-TB isolates, seven rifampicin-resistant isolates (based on GeneXpert data hence, potentially MDR-TB isolates), and 12 isoniazid mono-resistant isolates. Additionally, 40 isolates fully susceptible to both rifampicin and isoniazid were also investigated. Altogether, a total of 97 non-repetitive isolates were studied (one isolate per TB patient) and a control strain (H37Rv). S1 Table describes the characteristics of rifampicin and isoniazid resistant isolates investigated, while S1 Fig summarizes their genotypes (lineages/sub-lineages).

#### Rifampicin resistant isolates

Of the 45 rifampicin-resistant isolates, 69% (31/45) were *M*. *tuberculosis* lineage 4 (Euro-American), 24% (11/45) *M*. *tuberculosis* lineage 3 (East-African-Indian), and 7% (3/45) *M*. *tuberculosis* lineage 2 (East-Asian). The most prevalent sub-lineage among lineage 4 isolates was *M*. *tuberculosis* sub-lineage Uganda at 52% (16/31) with 35% (11/31) of these being sub-lineage Uganda II while 16% (5/31) were sub-lineage Uganda I. Altogether, lineage 4/sub-lineage Uganda accounted for 36% (16/45) of the rifampicin-resistant isolates with 24% (11/45) being Uganda II and 11% (5/45) Uganda I. The remaining sub-lineages combined accounted for 64% (29/45) of the rifampicin-resistant isolates and they included lineage 3/sub-lineage Delhi/CAS (24%, 11/45), lineage 2/sub-lineage Beijing (7%, 3/45), and lineage 4/sub-lineages LAM (11%, 5/45), Haarlem (11%, 5/45), Ghana (1/45, 2%), S (1/45, 2%), URAL (1/45, 2%), NEW-1 (1/45, 2%), and TUR (1/45, 2%), S1 Table and S1 Fig.

#### Isoniazid resistant isolates

Of the 50 isoniazid-resistant isolates, 72% (36/50) were *M*. *tuberculosis* lineage 4, 14% (7/50) *M*. *tuberculosis* lineage 3, and 14% (7/50) *M*. *tuberculosis* lineage 2. Similarly, the most prevalent sub-lineage among lineage 4 strains was *M*. *tuberculosis* sub-lineage Uganda at 56% (20/36), 47% (17/36) of which were Uganda II while 8% (3/36) were Uganda I. Overall, the predominant lineage 4/sub-lineage Uganda accounted for 40% (20/50) of the isoniazid-resistant isolates with 34% (17/50) being Uganda II and 6% (3/50) Uganda I. The remaining sub-lineages combined accounted for 60% (30/50) of the isolates and they included lineage 3/sub-lineage Delhi/CAS (14%, 7/50), lineage 2/sub-lineage Beijing (14%, 7/50), and lineage 4 sub/lineages LAM (10%, 5/50), Haarlem (10%, 5/50), Ghana (4%, 2/50), S (1/50, 2%), URAL (1/50, 2%), NEW-1 (1/50, 2%), and TUR (1/50, 2%), S1 Table and S1 Fig.

### Frequency of rifampicin- and isoniazid-resistance conferring mutations

#### *rpoB* and rifampicin-resistance

Sequence analysis of the *rpoB*/RRDR revealed mutations in six codons namely 588, 531, 526, 516, 513, and 511. The most frequent mutation was Ser531Leu (40%, 18/45), while the second most frequent mutations affected codon 526 (27%, 12/45) with various non-synonymous mutations i.e. His526Asp (6 isolates), His526Tyr (3 isolates), His526Arg (1 isolate), His526Gly (1 isolate), and His526Leu (1 isolate). The third most frequent mutation was Asp516Tyr (9%, 4/45) while the infrequent mutations were Leu511Pro (4%, 2/45), Glu588Gly (2%, 1/45), and Glu513Lys (2%, 1/45), Table 1 and S1 Table.

**Table 1:** Frequency and patterns of resistance conferring mutations in *M*. *tuberculosis* isolates that were resistant to rifampicin (n = 45) and isoniazid (n = 50).

Drug	Locus	Mutation^a^	Frequency (No. of Isolates)	Relative Frequency^b^ (%)
		Ser531Leu	18	18/45 (40)
		His526X^**c**^	12	12/45 (27)
RIFAMPICIN	*rpoB*	Asp516Tyr	04	4/45 (9)
		**Any of Ser531Leu, His526Tyr or Asp516Tyr**	**34**	**34/45 (76)**
		Leu511Pro	02	2/45 (4)
		Glu513Lys	01	1/45 (2)
		Glu588Gly	01	1/45 (2)
		**No mutation in rpoB**	**08**	**8/45 (18)**
		Ser315Thr	38	38/50 (76)
	*katG*	**Any of Ser315Thr, C(-15)T or T(-8)C**	**40**	**40 /50 (80)**
		Ser315Thr+any mutation in *inhA* gene promoter	06	06/50 (12)
** **		**No mutation in *katG***	**12**	**12/50 (24)**
		Any mutation in *inhA* gene promoter	08	08/50 (16)
		Any mutation in *inhA* gene promoter but no mutation in *katG* (Ser315Thr)	02 [with C(-8)A or C(-15)T]	02/50 (4)
		C(-15)T + Ser315Thr	04	04/50 (8)
		C(-15)T	04	4/50 (8)
ISONIAZID		T(-8)C	02	2/50 (4)
	*inhA* gene promoter	A(-7)C	1 (occurred in isolate with G(-116)A)	1/50 (2)
		G(-116)A	01	1/50 (2)
		A(-255)C	01	1/50 (2)
		C(-305)T	1 (occurred in isolate with C(-15)T)	1/50 (2)
		**No mutation in *inhA***	**42**	**42/50 (84)**
		**No mutation both in *inhA* & *katG***	**10**	**10/50 (20)**

^**a**^We used the Escherichia coli codon numbering for rpoB mutations

^**b**^Compared with the total number of isolates resistant to drug of interest see Campbell et al (2011) [6]

^**c**^Mutations affecting the 526 codon included His526Asp (6 isolates), His526Tyr (3 isolates), His526Arg (1 isolate), His526Gly (1 isolate), and His526Leu (1 isolate).

Overall, mutations affecting three codons 531, 526, and 516, which have been reported to be responsible for up to 90% of rifampicin-resistance in most settings, accounted for only 76% of the rifampicin-resistant isolates investigated. One isolate was a double mutant as it possessed both Asp516Val and Glu588Gly mutations, S1 Table. Interestingly, 18% (8/45) of the rifampicin-resistant isolates lacked mutations in the *rpoB*/RRDR, Table 1 and S1 Table. These isolates were subjected to the Genotype MTBDR*plus* assay as an extra quality control measure but still, we did not detect resistance conferring mutations in the *rpoB*/RRDR of those isolates.

#### *katG*/*inhA* gene promoter and isoniazid resistance

Sequence analysis of *katG* gene revealed Ser315Thr mutation as the most frequent at 76% (38/50) in isoniazid-resistant isolates (Table 1), and two of these isolates possessed both Ser315Thr and Asp406Ala (S1 Table). Furthermore, six mutations were identified in the *inhA* gene promoter namely C(-15)T (8%, 4/50), T(-8)C (4%, 2/50), A(-255)C (2%, 1/50), G(-116)A (*2*%, 1/50), A(-7)C (2%, 1/50), and C(-305)T (2%, 1/50). Overall, mutations in *katG* and the *inhA* gene promoter that are frequently associated with isoniazid-resistance in most settings [i.e. Ser315Thr and C(-15)T & T(-8)C, respectively] accounted for 88% of isoniazid-resistance, with *katG*/Ser315Thr alone accounting for 76% of the resistance. Nevertheless, 20% (10/50) of the isoniazid-resistant isolates lacked mutations in *katG* and *inhA* gene promoter, Table 1. These isolates were also re-tested with the Genotype MTBDR*plus* assay as an extra quality control measure and we confirmed absence of known isoniazid-resistance conferring mutations in those isolates. All the rifampicin- and isoniazid-resistant isolates that lacked resistance conferring mutations were MDR however, none of them simultaneously lacked mutations to both drugs, S1 Table.

#### No resistance conferring mutations in drug susceptible isolates

All the 40 isolates that were susceptible to rifampicin and isoniazid did not possess resistance conferring mutations in similar genes/loci analysed, implying that drug susceptible TB isolates in Uganda do not harbour mutations associated with resistance to rifampicin and isoniazid. Furthermore, the association between phenotypic rifampicin/isoniazid-resistance and Sanger DNA sequencing data was evaluated to determine the sensitivity of detecting rifampicin/isoniazid-resistance based on identification of high confidence resistance-conferring mutations. For rifampicin-resistance, the sensitivity of sequence analysis of *rpoB*/RRDR for the three mutations Ser531Leu, His526Tyr and Asp516Tyr was 81% (95%CI: 65.88%-91.40%). For isoniazid-resistance, the sensitivity of sequence analysis of both *katG* and *inhA* gene promoter for mutations Ser315Thr & C(-15)T and T(-8)C respectively, was 77% (95%CI: 63.16–87.47%).

#### Frequency of resistance conferring mutations in the circulating strains

Of the 18 rifampicin-resistant isolates that had the most frequent high confidence mutation *rpoB*/Ser531Leu, six (33%, 6/18) were lineage 3/sub-lineage Delhi/CAS and five (28%, 5/18) lineage 4/sub-lineage Uganda; the remaining seven isolates with the mutation *rpoB*/Ser531Leu were lineage 4/sub-lineage LAM (22%, 4/18) and lineage 2/sub-lineage Beijing (17%, 3/18), Table 2. Additionally, isolates that carried the two other commonly occurring high confidence for rifampicin-resistance (i.e. His526Tyr, Asp516Tyr) were lineage 3/sub-lineage Delhi/CASI, and lineage 4/sub-lineages TUR, URAL, and Haarlem. Though, three lineage 4/sub-lineage Uganda II isolates possessed His526Asp while a double mutant with Asp516Val and Glu588Gly was a Uganda II genotype isolate, Table 2.

**Table 2:** Frequency of rifampicin/isoniazid-resistance conferring mutations in the circulating *M*. *tuberculosis* genotypes/sub-lineages in Uganda (2009–2011).

	Uganda I	Uganda II	Delhi/CAS	Ghana	Beijing	LAM	NEW-1	Haarlem	URAL	TUR	S	Sub- total
**Rifampicin resistance**												
Ser531Leu	2	3	6	-	3	4	-	-	-	-	-	**18**
His526Asp	-	3	1	-	-	-	1	1	-	-	-	**06**
His526Tyr	-	-	1	-	-	-	-	-	1	1	-	**03**
Asp516Tyr	-	-	-	-	-	-	-	3	-	-	-	**03**
Leu511Pro	-	-	2	-	-	-	-	-	-	-	-	**02**
Asp516Val & Glu588Gly	-	1	-	-	-	-	-	-	-	-	-	**01**
Glu513Lys	1	-	-	-	-	-	-	-	-	-	-	**01**
His526Arg	-	-	-	-	-	1	-	-	-	-	-	**01**
His526Leu	-	-	1	-	-	-	-	-	-	-	-	**01**
His526Gly	-	-	-	-	-	-	-	1	-	-	-	**01**
No mutation	2	4	-	1	-	-	-	-	-	-	1	**08**
	**3**	**7**	**11**	**-**	**3**	**5**	**1**	**5**	**1**	**1**	**-**	
**Sub-total**												**45**
**Isoniazid resistance**												
*katG* mutations												
Ser315Thr	5	7	5	1	6	5	1	6	1	-	1	**38**
Ser315Thr + Asp406Ala	-	1	-	-	1	-	1	-	-	-	-	**03**
Ser315Thr + Insertion*	-	1	-	-	-	-	-	-	-	-	-	**01**
Ser315Thr + Asp296Glu	-	1	-	-	-	-	-	-	-	-	-	**01**
**Sub-total (per sub-lineage)**	5	7	5	1	6	5	1	6	1	-	1	**38**
No mutation	-	6	2	1	-	2	-	-	-	1		**12**
**Sub-total**												**50**
*inhA* promoter												
C(-15)T	1	2	1	-	-	-	-	-	-	-	-	**04**
C(-8)T	-	1	-	-	1	-	-	-	-	-	-	**02**
A(-255)C	-	-	-	-	-	1	-	-	-	-	-	**01**
G (-116)A	-	-	-	-	1	-	-	-	-	-	-	**01**
A(-7)C	-	-	-	-	1	-	-	-	-	-	-	**01**
C(-8)A	-	-	-	-	1	-	-	-	-	-	-	**01**
**Sub-total**	-	2	1	-	4	-	-	-	-	-	-	
**No mutation**	**-**	**-**	**-**	-	**-**	-	**-**	**-**	**-**	**-**	-	**42**

*Frame shift mutation; *insertion of Adenine at nucleotide position 845 of the katG gene*

Furthermore, of the 38 isoniazid-resistant isolates with the prevalent *katG*/Ser315Thr mutation, 12 (32%) were lineage 4/sub-lineage Uganda, of which seven were Uganda II (18%) and five Uganda I (13%). The remaining *katG*/Ser315Thr mutations were distributed as follows: 16% (6/38) each in lineage 4/sub-lineage Haarlem and lineage 2/sub-lineage Beijing; 13% (5/38) in lineage 3/sub-lineage Delhi/CAS, and 3% (1/38) each in lineage 4 sub-lineages S, Ghana, URAL, and NEW-1. Additionally, two isolates with *katG*/Asp406Ala mutation were lineage 4/sub-lineage NEW-1 and lineage 2/sub-lineage Beijing strains, S1 Table. Overall, *katG* mutations were most frequent in sub-lineage Uganda strains at 30% (12/50), followed by sub-lineages Beijing (12%, 6/50), Haarlem (12%, 6/50), LAM (10%, 5/50), and Delhi/CAS (10%, 5/50). Additionally, three of the four isoniazid-resistant isolates with the *inhA* gene promoter mutation C(-15)T were sub-lineage Uganda; likewise, one of the two isolates with the mutation T(-8)C was sub-lineage Uganda while the other was sub-lineage Beijing, S1 Table. The rare *inhA* gene promoter mutations A(-7)C, G(-116)A, & A(-255)C occurred in sub-lineages Beijing and LAM, respectively, Table 2.

Overall, the high confidence mutations for isoniazid-resistance (i.e. Ser315Thr, C(-15)T, T(-8)C) were more frequent than the high confidence mutations for rifampicin-resistance (i.e. Ser531Leu, His526Tyr, Asp516Tyr) in isolates with a genetic background of “*M*. *tuberculosis* Uganda” (P = 0.0298), S1 Table, perhaps explaining its weak association with MDR-TB. The other statistically significant associations between occurrence of resistance conferring mutations and the circulating *M*. *tuberculosis* genotypes in Uganda were (a) *rpoB*/Ser531Leu mutation being more prevalent in lineages 2 and 3 combined compared to lineage 4 strains (P = 0.0251), and (b) isoniazid-resistance conferring mutations Ser315Thr, C(-15)T, and C(-8)T being more prevalent in lineage 4 strains compared to lineage 2 strains (P = 0.0418), S1 Table. All the other noticed differences in association between occurrence of resistance-conferring mutations vis-à-vis circulating TB lineages/sub-lineages were not statistically significant, S1 Table.

## Discussion

In this study, we have demonstrated that sequence analysis of *rpoB*/RRDR, *katG* and *inhA* gene promoter is useful for the detection of rifampicin- and isoniazid-resistant *M*. *tuberculosis* isolates in Uganda. Indeed, all the susceptible isolates investigated lacked resistance conferring mutations implying that drug susceptible *M*. *tuberculosis* isolates in Uganda do not harbour mutations associated with resistance to rifampicin and isoniazid, although susceptible strains with these mutations do occur in other settings [6]. Importantly, we have shown that certain *M*. *tuberculosis* clinical isolates in Uganda are phenotypically resistant to rifampicin or isoniazid, but lack high confidence resistance-conferring mutations; these isolates do occur in Uganda at significantly high rates implying that rapid molecular tests like the Xpert MTB/RIF and Genotype MTBDR*plus* will not detect them as resistant. Also, we have shown that a comparatively low frequency of high confidence mutations for rifampicin-resistance in *M*. *tuberculosis* sub-lineage Uganda strains that are predominant in Uganda explains the previously reported weak association between MDR-TB and *M*. *tuberculosis* Uganda genotype [28, 29].

Among the 45 rifampicin-resistant isolates investigated, Ser531Leu was the most frequent mutation (40%). This mutation was previously reported at 70% prevalence by an earlier study in Uganda [20] that investigated ≤10 rifampicin-resistant isolates, and at 58% prevalence by another study in Uganda that investigated Xpert/MTB RIF data [7]. Although our findings agree with several other studies that reported predominance of Ser531Leu in rifampicin resistant *M*. *tuberculosis* isolates (e.g. 67.2% in Georgia [30] and Mexico [31], 58% Pakistan [32], 71% Germany [33], 59% Italy [34], 56% Greece [35], and 43% Japan [36], rates for Ser531Leu in Uganda are somewhat low, and confirm a previous prevalence of 41% (21/51) for this mutation in a study that used whole genome sequencing to investigate transmission of MDR-TB at Mulago National Referral Hospital in Kampala Uganda [37]. Overall, mutations affecting the three codons 531, 526 and 516 combined, which are frequently associated with rifampicin-resistance in most settings, accounted for 76% of the rifampicin resistance in this study. The second most frequent mutation His526Asp, accounted for 13% of the rifampicin resistant isolates. This mutation was previously reported in Uganda [13, 20] though at a lower frequency. Indeed, codon 526 in *rpoB*/RRDR of rifampicin resistant isolates in this study possessed several non-synonymous mutations i.e. His526Asp (6 isolates), His526Tyr (3 isolates), His526Arg (1 isolate), His526Gly (1 isolate), and His526Leu (1 isolate). Our findings for codon 526 are in agreement with the findings of Clarke et al (2013), who also reported diverse mutations at codon 526 i.e. His526Asp (3 isolates), His526Arg (1 isolate), His526Tyr (2 isolates), and His526Asn (1 isolate) [37] in MDR-TB isolates at Mulago Hospital. His526Tyr is a well-documented high confidence mutation for rifampicin-resistance in several settings, alongside Ser531Leu and Asp516Val [6, 25]. Overall, there is variation in frequencies of mutations at codon 526 (especially His526Tyr vs. His526Asp) in rifampicin resistant *M*. *tuberculosis* isolates from various settings, and we have shown that His526Asp is comparatively more frequent in isolates from Uganda. Note, some of the identified mutations at codon 526 e.g. His526Leu had not been reported before in isolates from Uganda.

Furthermore, only four (8.9%, 4/45) rifampicin-resistant isolates possessed Asp516Tyr, the third most frequent high confidence mutation for rifampicin-resistance; again, our findings are comparable with Clarke et al (2013) who reported five (9.8%, 5/51) MDR-TB isolates with mutations at codon 516 at Mulago Hospital [37]. The Asp516Tyr mutation was previously described by other investigators in Uganda [13, 20], also at low frequencies. Three isolates with Asp516Tyr were lineage 4/sub-lineage Haarlem strains, in accordance with previous reports from Spain [38]. Importantly, an isolate with the Asp516Val mutation also had Glu588Gly (double mutant); Glu588Gly has not been reported before in Uganda but was documented elsewhere in a survey that compared INNO-LiPA and MTBDR*plus* tests for identification of *rpoB* point mutations [39]. Another lineage 4/sub-lineage Haarlem isolate possessed His526Gly, a unique mutation given that it is generated by substitution of two bases at nucleotide positions 1333 and 1334. This mutation has not been reported in many settings including Uganda though it was previously associated with rifampicin-resistance [40]. Furthermore, two isolates had Leu511Pro, a mutation that was previously reported to occur in rifampicin-resistant *M*. *tuberculosis* from Uganda at 8% prevalence [7]. Elsewhere, Leu511Pro was designated a high confidence mutation in rifampicin resistant *M*. *tuberculosis* [41], and it has been reported among rifampicin resistant *M*. *tuberculosis* isolates in the UK [42].

For isoniazid, only 38 (76%) of the resistant isolates possessed the *katG/*Ser315Thr mutation, a finding comparable with reports from many settings worldwide [6, 18] including Mulago National Referral Hospital in Kampala i.e. 58.8% (30/51) frequency for *katG*/Ser315 [37]. *katG/*Ser315Thr was found frequent in isoniazid resistant isolates in Belarus (100%), China (55%), Honduras (60%), Iran (40%), Romania (80%) [20], Nepal (62.2%) [43] and Poland (72%) [44]. As such, *katG*/Ser315Thr is a reliable target for rapid detection of isoniazid resistant *M*. *tuberculosis* irrespective of geographic location. However, significant variation by geographic region in its distribution has been reported [18]; for instance, of the 808 isoniazid resistant isolates from Japan and Korea, only 43% had *katG*/Ser315Thr [18]. In contrast, this mutation was observed in 94% of the 751 isoniazid resistant isolates from Eastern Europe. Importantly, sub-Saharan Africa was poorly represented in this analysis and a need for systematic surveys of regional patterns in mutations in the sub-continent was highlighted [18].

Furthermore, an isoniazid resistant isolate in this study had a frame shift mutation resulting from insertion of adenine at position 845 base pairs, which could have inactivated *katG*. Thus, isoniazid-resistance in this sole isolate with a frame shift mutation was probably due to deficiency of catalase peroxidase enzyme that activates isoniazid [6, 45, 46]. *M*. *tuberculosis* clinical isolates with deletion of the *katG* gene, which is required for catalase-peroxidase activation of isoniazid, were reported previously for two MDR-TB isolates in Uganda [37]. In addition, two isolates possessed *katG/*Asp406Ala, a mutation that substitutes a polar amino acid Aspartate with an aliphatic one, Alanine, which may lead to expression of a modified catalase peroxidase leading to insufficient isoniazid breakdown. The mutation Asp406Ala in *katG* has not been described in previous studies of isoniazid-resistant *M*. *tuberculosis* isolates in Uganda.

Within the *inhA* gene promoter, only 4 (8%) isoniazid-resistant isolates possessed the C(-15)T mutation. This finding is comparable with results of a study that reported a prevalence of 5% for this mutation in MDR-TB isolates from Uganda [20]. The C(-15)T mutation is associated with low level resistance to isoniazid [47] and it occurs at lower frequencies compared to *katG/*Ser315Thr [6, 18]. Further, two isolates possessed T(-8)C, a mutation that is also associated with low level resistance to isoniazid. This mutation was reported previously in MDR-TB isolates from Uganda, Romania, Honduras, Iran, and Belarus, also at low frequencies [20]. Other *inhA* gene promoter mutations [A(-255)C, G(-116)A, A(-7)C, C(-8)A] occurred at much lower frequencies compared to C(-15)T and T(-8)C and this is consistent with findings from other investigators [18, 40], implying that isoniazid-resistance in Uganda is mostly attributable to the *katG*/Ser315Thr mutation. Indeed, majority (32, 78%) of the isoniazid-resistant isolates that lacked mutations in *inhA* gene promoter harboured *katG*/Ser315Thr, and they could still be detected by the rapid molecular assays as they target mutations both in *katG* and *inhA* promoter [47].

An unusual finding in this study was the relatively high prevalence of rifampicin- and isoniazid- resistant *M*. *tuberculosis* isolates that lacked resistance conferring mutations in *rpoB*/RRDR, *katG* and *inhA* promoter. However, similar findings were reported from other TB endemic countries [48, 49]. In fact, MDR-TB isolates that are phenotypically resistant to rifampicin (three isolates) and isoniazid (five isolates) but lacking resistance conferring mutations have already been reported in isolates from Uganda [37]. Yet, we have reported higher prevalence of those isolates in Uganda, implying they could prove to be problematic in the management of drug resistant TB patients. Further studies are required to fully characterize the molecular epidemiology of these isolates in Uganda, especially studies employing high throughput sequencing approaches to uncover all the potential resistance conferring mutations. Since the commercial rapid molecular assays for TB target only high confidence mutations to swiftly detect resistance to anti-TB drugs [31, 32, 42, 50], absence of targets is troublesome as it makes it nearly impossible to employ rapid molecular assays to detect certain resistant strains in clinical samples. On the other hand, strains that lacked mutations could harbour resistance conferring mutations in other genes e.g. *ahpC*, *oxyR*, *kasA*, *furA* and *ndh* that are associated with isoniazid-resistance [18, 51], or the use of efflux pumps for rifampicin-resistance [20, 25] but we did not investigate these mechanisms. Furthermore, although the resistant isolates that lacked known-resistance conferring mutations were validated as having no mutations, hetero-resistance due to mixed-strain resistant-plus-susceptible TB strains might have confounded the results, as both Sanger-DNA sequencing and rapid molecular assays (i.e. GeneXpert and LPAs) do not detect mixed-strain tuberculosis infections. In theory, mixed-strain tuberculosis infections can negatively impact the performance of rapid molecular tests for TB, especially in samples with hetero-resistant strains. Nevertheless, drug resistant strains lacking known resistance conferring mutations to rifampicin and isoniazid do occur [6], and our detection of these isolates is in agreement with several other global studies that described them [6, 18, 52–54].

### Limitations

Our study focused on targeted Sanger-DNA sequencing of specific genes/loci associated with rifampicin and isoniazid resistant *M*. *tuberculosis* and this may not reveal all the possible mutations associated with rifampicin- and isoniazid-resistance. However, as a limited number of mutations is associated with resistance to rifampicin [6] and isoniazid [18], it is likely we covered the major mutations that confer resistance to these drugs in Uganda.

## Conclusions

Sequence analysis of *rpoB*/RRDR, *katG* and *inhA* gene promoter is useful in the detection of rifampicin/isoniazid-resistant *M*. *tuberculosis* isolates in Uganda, and has revealed that drug susceptible isolates in Uganda do not harbour mutations associated with resistance to rifampicin and isoniazid. The most prevalent high confidence mutation for rifampicin-resistance in Uganda is *rpoB*/Ser531Leu, although its frequency is low compared to estimates from other countries. Accordingly, a comparatively low frequency of high confidence mutations for rifampicin-resistance in *M*. *tuberculosis* sub-lineage Uganda strains explains their weak association with MDR-TB. Furthermore, *katG*/Ser315Thr is the most frequent high confidence mutation for isoniazid-resistance, and its frequency is comparable to estimates from other countries. Overall, a significant number (≤20%) of rifampicin/isoniazid-resistant isolates in Uganda lack high confidence resistance conferring mutations, implying that the commercial rapid molecular assays (e.g. Xpert MTB/RIF, Genotype MTBDR*plus*) will not detect them as resistant, potentially complicating the control of MDR-TB.

## Materials and methods

### Ethics approval

This study was approved by the Higher Degrees Research and Ethics Committee of Makerere University School of Biomedical Sciences. This committee authorized the use of archived *M*. *tuberculosis* isolates from adult patients ≥18 years who participated in the National and Kampala Drug Resistance Surveys that investigated phenotypic resistance to first- and second-line anti-TB drugs among new and previously treated TB cases in Uganda [55, 56]. Patient data was excluded from the characterized bacterial isolates however, the parent studies [55, 56] obtained written informed consent from the participants to participate in the study, and consent for sample storage and use of the stored sputum samples in further studies.

### Study design and setting

This was a retrospective cross-sectional laboratory-based study conducted at the Molecular Biology Laboratory, Department of Immunology and Molecular Biology, Makerere University College of Health Sciences. The *M*. *tuberculosis* isolates investigated were collected during the recent Kampala and National TB Drug Resistance Surveys [55, 56]. Demographic data, sputum sample collection and culturing were described previously [55, 56]. Also, phenotypic drug susceptibility testing (DST) of the isolates was described previously [55, 56] but repeat testing was performed to validate the susceptibity patterns. As samples were taken from a nationwide cohort of new and previously treated sputum smear-positive TB patients registered at TB diagnostic centres in Uganda [55], the investigated isolates are representative of the entire country.

In the current study, we included 50 isoniazid-resistant isolates, 38 of which were also rifampicin-resistant (MDR isolates) while 12 were isoniazid-mono resistant. Additionally, 45 rifampicin-resistant isolates were studied, of which 38 were MDR while seven were rifampicin-resistant based on GeneXpert alone and lacked phenotypic DST data for isoniazid and rifampicin at the time of analysis. For quality control, 40 *M*. *tuberculosis* isolates that were fully susceptible to rifampicin, isoniazid and other first-line anti-TB drugs, were similarly investigated. Altogether, a total of 97 non-repetitive isolates (one isolate per patient) and a H37Rv laboratory strain were investigated.

### Recovery and harvesting of the isolates

*M*. *tuberculosis* culturing and manipulation were performed in a BSL-3 Mycobacteriology Laboratory of the Dept. of Medical Microbiology, Makerere University College of Health Sciences. Briefly, the isolates were recovered by sub-culturing on Middlebrook 7H10 agar (Becton and Dickson, USA), incubating at 37°C in a CO_2_ incubator (Thermal Scientific, USA), and observing daily for growth of the organisms for 28 days. The cells were harvested and re-suspended in absolute ethanol (Sigma scientific, USA) to kill them by suffocation. The suspension was centrifuged at 16,000g to obtain the cell pellet that was later re-suspended in 0.25X Tris-EDTA (TE) buffer.

### Chromosomal DNA extraction

High quality chromosomal DNA was extracted from *M*. *tuberculosis* by following the CTAB/chloroform extraction method [57]. Briefly, the cells, suspended in 0.25X TE buffer, were centrifuged at 3,000 g to wash off the media salts and residual ethanol. To lyse the cells, the cell pellet was re-suspended in 400 μL of freshly prepared 0.25X TE buffer, to which 50 μL of Lysozyme (40 mg/mL) was added and the mixture incubated at 37°C, overnight. Then, 150μL of 10% SDS/Proteinase K solution was added to the mixture and incubated at 65°C for 1 hour to ascertain complete cell lysis, precipitation of proteins and other cell debris. After this, 100μL of 5M NaCl was added to each tube followed by addition of 100μL of CTAB/NaCl and inverting the mixture several times until the content turned milky. To ensure complete precipitation of cell debris the solution was incubated at 65°C for 10 min. To purify the DNA, 750 μL of Chloroform:Isoamyl alcohol mixture was added to the samples, followed by centrifugation at 16,000 g for 10 min. After this, the aqueous phase that contained the DNA was carefully transferred to a pre-labelled sterile 1.5ml centrifuge tube (Eppendorf Inc.), to which 600μL of absolute ice cold isopropanol (Fisher Scientific, USA) was added to precipitate the genomic DNA. After incubating at -20°C for 2 hours, the mixture was centrifuged for 10 min at 16,000 g to pellet the DNA. The pellet was washed with 1ml of 70% ice-cold ethanol and dried at room temperature for 1 hour. The DNA was eluted in 50μL of 0.25X TE buffer. Prior to use as template in PCRs the quality and quantity of the extracted DNA was determined by electrophoresis on a 1% agarose gel and a NanoDrop spectrophotometer (ThermoFisher Scientific).

### Genotyping

Isolates were confirmed as belonging to the *M*. *tuberculosis* complex (MTBC) by using an in-house PCR protocol described previously [58], in which they all tested positive for the insertion sequence *IS6110* confirmatory for MTBC. Furthermore, 15 loci Mycobacterial Interspersed Repetitive Units-Variable Number of Tandem Repeats (MIRU-VNTR) analysis was performed as described previously [59] to determine the *M*. *tuberculosis* genotypes/sub-lineages.

### PCR, DNA-sequencing and analysis

We used previously published gene/locus specific primers and procedures to amplify specific fragments of rifampicin- and isoniazid-resistance associated genes/loci (i.e. *rpoB*, *katG*, *inhA* gene promoter) from *M*. *tuberculosis* [6, 20]. The total reaction volume was 60μL, prepared according to the HotStar PCR kit protocol (Qiagen, Hilden, Germany). Briefly, each reaction contained 27.5μL nuclease free water, 6μL 10x PCR buffer, 12μL Q-solution, 3μL 10mM MgCl_2_, 3μL 10mM dNTPs, 1.5μL each of reverse and forward primers, 0.5μL High fidelity Taq DNA polymerase (5U/μL) (Sigma-Aldrich, USA), and 10ng/μL (in 5μL volume) chromosomal DNA template. Amplification was achieved in a BioRad T100 Thermal cycler (Bio-Rad Laboratories Inc., Singapore) using the following program: initial denaturation at 95°C, 5 min followed by 35 cycles each consisting of 95°C, 45 sec; 60°C, 45 sec; and 72°C, 50 sec with a final extension step of 72°C, 10 min. Five microliters each of the PCR product was analysed on a 1% agarose gel stained with ethidium bromide (0.5 μg/ml). Gels were run at 120V for 1 hour and visualized using a UVP Gel documentation (Benchtop Trans-illuminator System_BioDoc-it, CA, USA).

Furthermore, 50μL each of the PCR products was purified with the QIAmp DNA purification mini kit (Qiagen, Hilden, Germany), and the purified amplicons sequenced at ACGT Inc. (Wheeling IL, USA). The sequences we obtained (S2 Fig) were analysed first through BLAST searching at NCBI https://blast.ncbi.nlm.nih.gov/Blast.cgi to confirm they hit the expected gene sequences in *M*. *tuberculosis*. Poor quality bases at the beginning and end of the sequences were trimmed off. To determine the amino acid substitutions, DNA sequences were translated into amino acid sequences using the Molecular Evolutionary Genetics Analysis Version 6.0 (MEGA6.06) software [60] or the ExPASy-Translate server http://web.expasy.org/translate/. To determine presence/absence of mutations in the sequenced genes/loci, the curated sequences were aligned using MEGA6.06 (or BioEdit v7.2.5.0) to sequences of *rpoB*, *katG*, and *inhA* gene promoter obtained from the genome sequence of *M*. *tuberculosis* strain H37Rv (NCBI reference sequence NC_000962). To determine whether the identified mutations were resistance conferring, we used tbdream database [27] https://tbdreamdb.ki.se/Info/ and published literature [6, 18, 61, 62]. The data was curated, compiled and presented as tables or percentages depending on frequencies of mutations that occurred in the examined genes/loci (S1 Table). The Chi-square test was used to evaluate the associations between *M*. *tuberculosis* lineages, sub-lineages/genotypes and drug-resistance conferring mutations.

### Quality control

The *M*. *tuberculosis* laboratory strain H37Rv is susceptible to all anti-TB drugs and it was used as the wild-type in determining resistance-conferring mutations. Additionally, a known MDR-TB strain with well-characterized rifampicin/isoniazid-resistance conferring mutations was included as the positive control for resistance mutations. Negative controls for DNA extraction and PCRs were included to rule out contamination. High-Fidelity Taq DNA polymerase (Sigma-Aldrich, USA) that delivers superior results with twofold higher yield and threefold greater fidelity compared to regular Taq Polymerases was used in all the PCRs. All sequenced amplicons were confirmed through nucleotide-BLAST, translated-BLAST, and protein-BLAST analysis and they all hit the expected genes/loci in *M*. *tuberculosis*.

## Supporting information

S1 Table Characteristics of rifampicin and isoniazid resistant *M*. *tuberculosis* isolates investigated. (XLSX)

S1 Fig Panels A and B, genotypes/sub-lineages of *M*. *tuberculosis* isolates resistant to isoniazid and rifampicin, respectively. (TIFF)

S2 Fig Selected chromatograms for sequenced *rpoB*, *katG* and *inhA* gene promoter fragments from rifampicin/isoniazid resistant *M*. *tuberculosis* isolates. Codons of interest are circled. Panels: A, positive control (rifampicin-susceptible isolate) with wild-type codon TCG (encodes Serine at nucleotide position 531 of *rpoB/*RRDR; B, rifampicin-resistant isolate showing a transition at codon 531 in *rpoB*/RRDR that altered TCG to TTG (encodes Leucine), the most prevalent high confidence mutation (Ser531Leu) for rifampicin resistance; C & D, isoniazid-susceptible and isoniazid-resistant isolates, respectively, showing transversion (for resistant isolates) at *katG*/315 (AGC to ACC) that substituted Serine for Threonine hence the frequent high confidence mutation *katG*/Ser315Thr; E & F, isoniazid-susceptible and isoniazid-resistant isolates, respectively, showing mutation of C to T (resistant isolates) in *inhA* gene promoter at nucleotide position -15.
(TIFF)
