# Supplementary figures and images for: Prevalence and patterns of rifampicin and isoniazid resistance conferring mutations in Mycobacterium tuberculosis isolates from Uganda
Source: PLoS One. 2018 May 30;13(5):e0198091. doi: 10.1371/journal.pone.0198091 (PMC5976185; doi:10.1371/journal.pone.0198091)

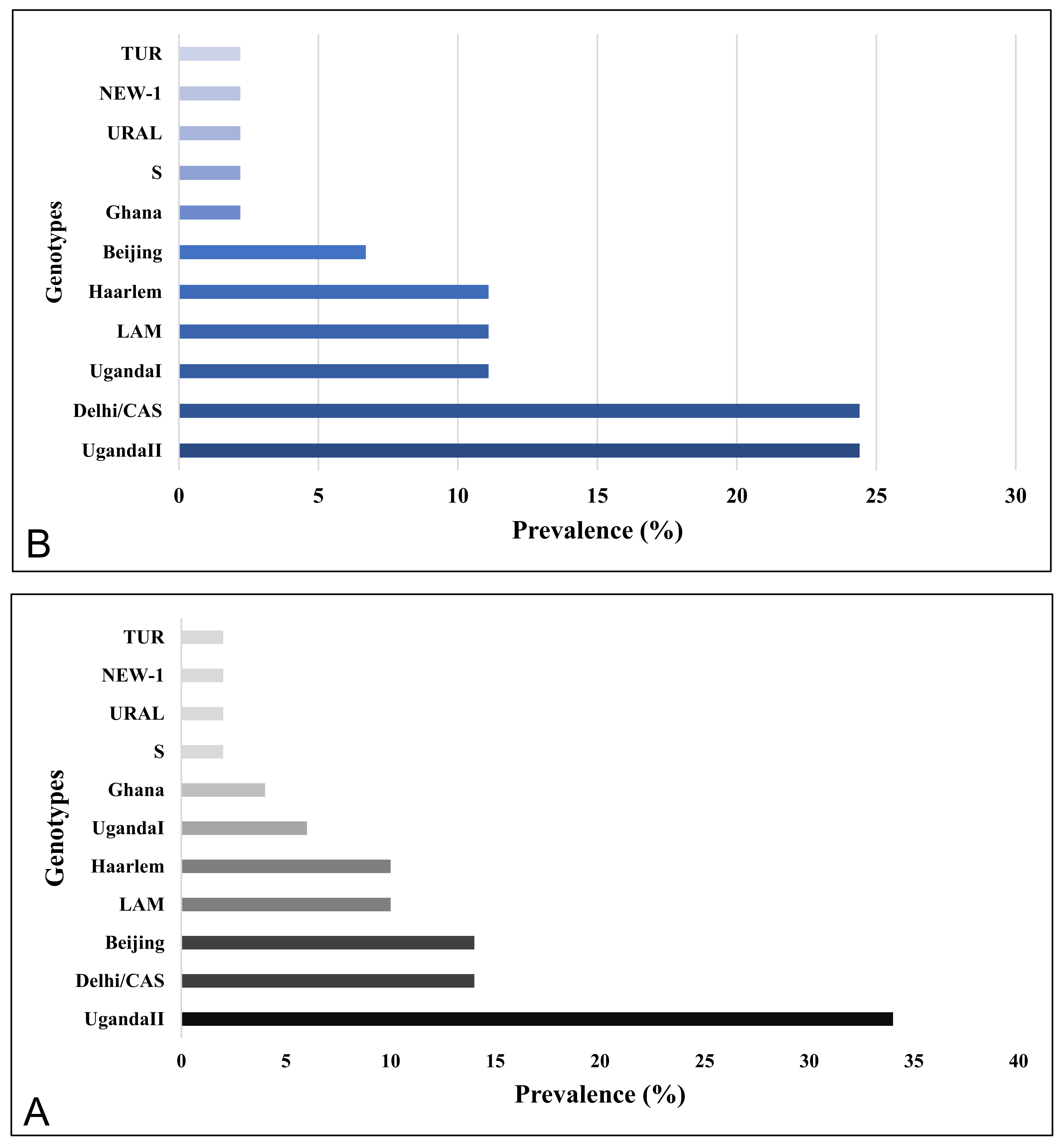

Supplement: S1 Fig — (TIFF) [file pone.0198091.s002.TIFF]

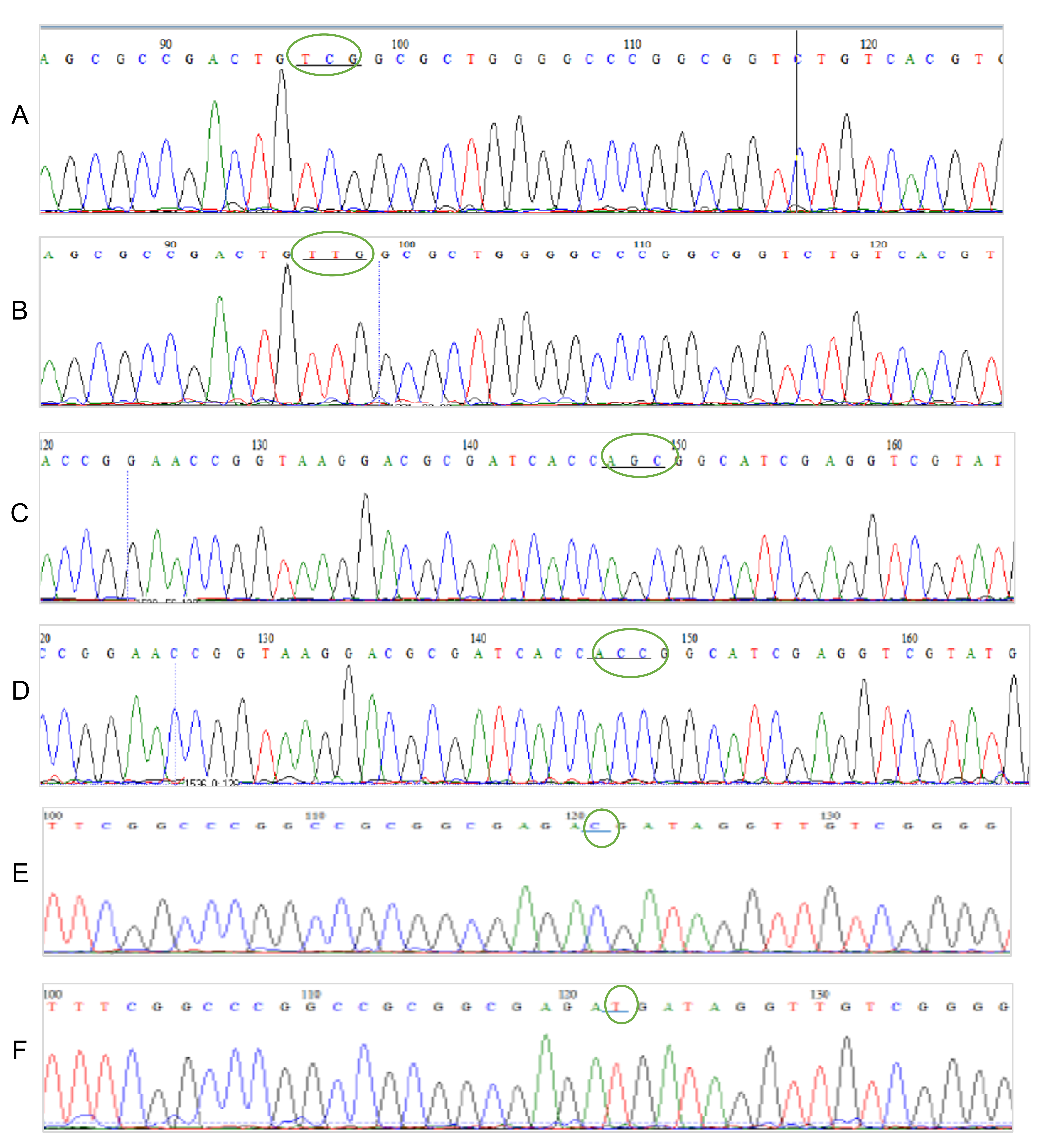

Supplement: S2 Fig — Codons of interest are circled. Panels: A, positive control (rifampicin-susceptible isolate) with wild-type codon TCG (encodes Serine at nucleotide position 531 of rpoB/RRDR; B, rifampicin-resistant isolate showing a transition at codon 531 in rpoB/RRDR that altered TCG to TTG (encodes Leucine), the most prevalent high confidence mutation (Ser531Leu) for rifampicin resistance; C & D, isoniazid-susceptible and isoniazid-resistant isolates, respectively, showing transversion (for resistant isolates) at katG/315 (AGC to ACC) that substituted Serine for Threonine hence the frequent high confidence mutation katG/Ser315Thr; E & F, isoniazid-susceptible and isoniazid-resistant isolates, respectively, showing mutation of C to T (resistant isolates) in inhA gene promoter at nucleotide position -15. (TIFF) [file pone.0198091.s003.TIFF]
